# Smartphone-Based Peptide Nucleic Acid (PNA) Probe-Assisted Potentiometric Biosensor for Point-of-Care Testing of SARS-CoV‑2 Nucleic Acid

**DOI:** 10.1021/acs.analchem.6c00766

**Published:** 2026-05-05

**Authors:** Tugba Ozer, Sakda Jampasa, Atchara Lomae, Nipapan Ruecha, Osman Sagdic, Tirayut Vilaivan, Charles S. Henry, Orawon Chailapakul

**Affiliations:** † Department of Bioengineering, Faculty of Chemical-Metallurgical Engineering, Yildiz Technical University, Istanbul 34220, Türkiye; ‡ Department of Chemistry, 416008 Colorado State University, Fort Collins, Colorado 80523, United States; § Futuristic Science Research Center, School of Science, 65133 Walailak University, Nakhon Si Thammarat 80160, Thailand; ∥ Center of Excellence for Trace Analysis and Biosensor, 26686 Prince of Songkla University, Songkhla 90110, Thailand; ⊥ Division of Physical Science, Faculty of Science, Prince of Songkla University, Songkhla 90110, Thailand; # Metallurgy and Materials Science Research Institute, 251501 Chulalongkorn University, Bangkok 10330, Thailand; g Department of Food Engineering, Faculty of Chemical-Metallurgical Engineering, Yildiz Technical University, Istanbul 34220, Türkiye; h Organic Synthesis Research Unit, Department of Chemistry, Faculty of Science, Chulalongkorn University, Bangkok 10330, Thailand; j Electrochemistry and Optical Spectroscopy Center of Excellence (EOSCE), Department of Chemistry, Faculty of Science, Chulalongkorn University, Bangkok 10330, Thailand

## Abstract

Herein, we present a potentiometric biosensor based on amine magnetic beads functionalized with electrostatically neutral peptide nucleic acids (PNA) for the detection of severe acute respiratory syndrome coronavirus 2 (SARS-CoV-2). A screen-printed carbon electrode was modified with a plasticized polymeric membrane. A SARS-CoV-2 specific peptide nucleic acid probe was designed and synthesized. We exploited the advantage of neutral PNAs to remove the negatively charged DNA probes that cause fouling due to the attraction of positively charged biomolecules. After specific binding of peptide nucleic acid to its complementary DNA target, the peptides were incubated with cationic magnetic nanoparticles. The potentiometric responses were recorded after applying a magnetic field for the rapid and sensitive detection of SARS-CoV-2. Various parameters including polymeric membrane composition, probe concentration and incubation time, amount and incubation duration of magnetic nanoparticles, the order of probe-target magnetic bead incubation, and the force of magnetic field effect were investigated. Under optimized conditions, SARS-CoV-2 DNA was detected in the linear range of 1.0 × 10^–15^–1.0 × 10^–11^ M with an LOD of 4.9 × 10^–16^ M. Moreover, the sensing performance of the developed biosensor in human saliva was evaluated, and the recovery rates were found to be 96–98%. For proof of concept, a smartphone-based near-field communication electrochemical analyzer was integrated into the developed biosensor for potentiometric measurements. This biosensor is promising, as the antifouling PNA probes provide a new concept for the development of sensitive biosensors for screening for COVID-19 in biological samples.

## Graphical abstract

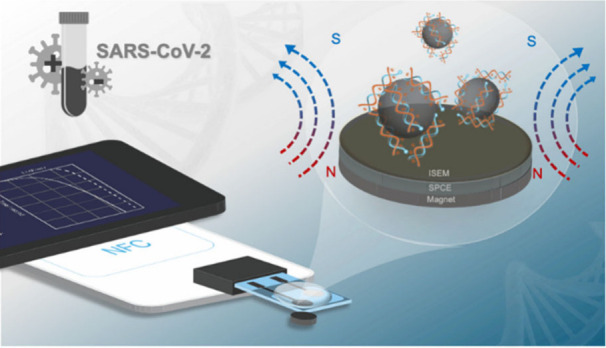

The COVID-19 pandemic has affected billions of people worldwide, resulting in over a million deaths and a global economic crisis which was more significant than the Second World War.^1^ Identification of the disease-causing virus SARS-CoV-2 is critical to monitor in individuals who may be infected and need direct treatment. Since SARS-CoV-2 is transmitted from presymptomatic, symptomatic, or asymptomatic individuals, decreasing virus spread becomes challenging by the identification of infected subjects using symptoms alone.^2^ Furthermore, influenza and COVID share common symptoms but different treatments. According to the Centers for Disease Control and Prevention (CDC), SARS-CoV-2 nucleic acids in oropharyngeal/nasopharyngeal (OP/NP) fluids could be accurately detected via a real-time reverse transcription-polymerase chain reaction (RT-PCR) to assess infection status.^3^ Although RT-PCR-based diagnostic kits are currently available for the detection of SARS-CoV-2, they have drawbacks that they require time-consuming analysis steps, expensive equipment, and skilled staff to operate as well as several hours for analysis.^4,5^ Due to the high operating costs of SARS-CoV-2 screening, population-wide diagnosis of COVID-19 remains limited in developing countries.^6^ Therefore, there is a need for point-of-care (POC) analytical devices for both symptomatic and asymptomatic individuals to rapidly and affordably diagnose an infection.

Owing to recent advancements in the fields of biosensors, there are various strategies that hold promise for the precise diagnosis of viral infections. In readily available COVID-19 test kits, antibodies such as immunoglobulins G (IgG) and M (IgM) have been widely used as specific biomarkers indicating COVID-19 onset; however, misdiagnosis can occur because of the lack of immunoglobulins in the patient’s blood during early stages of the disease because antibodies require at least 2 weeks to reach detectable levels, delaying treatment.^7,8^ Alternatively, nucleic-based assays where the complementary DNA receptors are used can selectively detect SARS-CoV-2.^9^ However, unlike DNA, RNA is easily degraded during the transportation and/or storage of samples.^10^

In contrast to the negatively charged DNA probe, the electrostatically neutral PNA probe does not interact nonspecifically with charged molecules or surfaces, thus offering new modes of DNA detection.^11,12^ Furthermore, PNA is considered to be a promising probe for target detection due to its distinctive properties such as stability and specificity.^13,14^ In addition, the use of PNA probes in DNA biosensors has been shown to have fast hybridization kinetics that are independent of ionic strength and require shorter probe lengths than DNA, further making it especially suitable for DNA sequence determination.^15^

Electrochemical biosensors offer sensitive, quantitative, and fast clinical outcomes at a reduced analysis cost. In particular, potentiometry is a well-established method that has been widely used for monitoring various analytes. Potentiometric ion-selective electrodes (ISEs) consist of a two-electrode system including a reference electrode and a working electrode.^16,17^ Among ISEs, solid-contact ISEs offer advantages over conventional ISEs due to their simplicity, ease of miniaturization, flexibility, and robustness.^18,19^ Paper as a substrate has been used to fabricate low-cost solid-contact ISEs; however, we recently reported that unmodified paper is problematic due to the presence of electrostatic interactions between functional groups on the paper substrate and the ions in the analyte.^20^

Here, we present a simple, robust, solid-contact-ISE-based screen-printed electrode planar platform assisted by magnetic nanoparticle-modified PNA as the probe along with a miniaturized NFC-based detector for the potentiometric detection of SARS-CoV-2. At first, a screen-printed electrode array was fabricated using graphite with a particle size of ∼30 μm and carbon ink on a transparent sheet as the substrate. Then, the working electrode surface was coated with a polymeric membrane whereas the reference electrode zone was coated with Ag/AgCl ink and the reference electrode membrane.^21,22^ For the detection probe, amine-functionalized cationic magnetic nanoparticles, exploiting the main benefits of nanomaterials including their higher surface area/volume ratio and magnetic properties, were biofunctionalized with PNA. The conformationally constrained acpcPNA which offers better affinity and specificity was used instead of conventional PNA.^23^ The optimization studies were performed to define the best membrane composition and volume of the membrane to obtain an improved potential signal during electrochemical measurements. In addition, all assay parameters, including magnetic bead concentration and incubation time, PNA concentration and hybridization time, and the magnetic force, were systematically optimized. Finally, the developed solid-contact ISE was used in combination with magnets and a smartphone to detect SARS-CoV-2 DNA in real samples. The developed sensing platform has advantages including low cost, portability, large-scale production, simplicity, disposability, the need for a small volume (microliters) of biofluids, and accurate analysis results on a smartphone.

## Experimental Section

### Aminated Magnetic Beads-acpcPNA Probe-DNA Hybridization on the Device

A solution with a final concentration of 10 μM PNA and 10 μM DNA in 10 mM PBS at pH 7.4 was prepared in an Eppendorf tube and incubated for 1 h with occasional vortex mixing to ensure mixing during the early stages of the optimization studies. MBs were prepared by dispersing 1 mg of aminated magnetic beads in 1 mL of 10 mM PBS at pH 7.4, followed by a 5 min of agitation and subsequent removal of the supernatant with the use of a magnet. The washing process was repeated three times. The washed MBs were resuspended in the solution of PNA-DNA assembly during a 1 h agitation. The supernatant was next removed, and the MBs were washed three times with 10 mM PBS at pH 7.4 using a magnet. After washing, the MB-PNA-DNA assembly was used for subsequent detection experiments immediately. In addition, the optimum conditions for acpcPNA-DNA hybridization and aminated magnetic bead interaction were investigated under the following conditions: (1) acpcPNA-DNA hybridization then mix with MBs, (2) MB-DNA complex formation then mix with acpcPNA, and (3) MB-acpcPNA complex formation then mix with DNA. In each case, the resultant suspension was separated using magnetic force in the presence of a magnet and washed with PBS buffer three times for further experiments.

To obtain the calibration curve of the developed biosensor, MB-acpcPNA and DNA at different concentrations (1.0 × 10^–11^–1.0 × 10^–15^ M) were incubated for 1 h with shaking using vortex mixing at room temperature. Then, 10 μL of the final solution was drop-cast onto the electrodes, and the potential changes of the polymeric membrane were recorded using an open circuit potential. The potential differences (Δ*E*) was obtained by subtracting the potential of the blank (probe-functionalized magnetic beads in PBS without target DNA) from the potential measured after incubation with the target DNA. Experimental parameters included the MB concentration, the incubation time, the acpcPNA concentration, the hybridization time, and the probe-target-MB incubation order. The incubation times were optimized sequentially after optimization of the other experimental parameters. Initially, magnetic beads were incubated with the PNA probe for 2 h, followed by a 2 h DNA hybridization step.

In addition, the magnetic force applied during the potentiometric measurements was controlled using neodymium permanent magnets with the use of a varying number of magnets. Each magnet had a diameter of 3 mm and a thickness of 0.8 mm, with a surface magnetic field strength of approximately 295.2 mT (K&J Magnetics). The magnets were positioned directly beneath the working electrode. The magnetic force was varied by changing the number of magnets placed beneath the electrode (1–4 magnets stacked vertically) while maintaining the same alignment and distance relative to the electrode surface. This approach allowed modulation of the magnetic attraction exerted on the magnetic beads during the potentiometric measurements.

## Results and Discussion

### Working Principle of the Biosensor

This study aims to explore a novel principle for the potentiometric detection of SARS-CoV-2. As shown in Figure 1, our potentiometric DNA-based platform features the sensing performance of magnetopotentiometry with a polymeric membrane-modified disposable screen-printed electrode (SPE). The electrostatically neutral acpcPNA probe that shows a unique DNA recognition ability^24^ was employed as a recognition element, whereas the cationic aminated magnetic beads were electrostatically attracted to the acpcPNA-DNA complex via the negative charge of the DNA and the positive charge of the amino groups on the MB surface.^25^ A screen-printed potentiometric sensor consisting of a solid-state Ag/AgCl reference electrode and a polymeric membrane modified working electrode with magnets on the back side was employed for the detection of COVID-19 DNA in saliva samples. The polymeric membrane–sample solution interface is controlled by the magnetic properties of the aminated MB-acpcPNA-DNA complex. Accumulation of the complex on the electrode surface was facilitated by magnetic fields. The resulting change in the phase-boundary potential, arising from ion-exchange processes at the polymeric membrane interface, generates a potentiometric signal upon target binding. Furthermore, the hydrophilic nature of the phosphate-rich DNA and acpcPNA molecules facilitates the efficient extraction of the acpcPNA-DNA complex into the polymeric membrane under the influence of the magnetic field.

**Figure 1:**
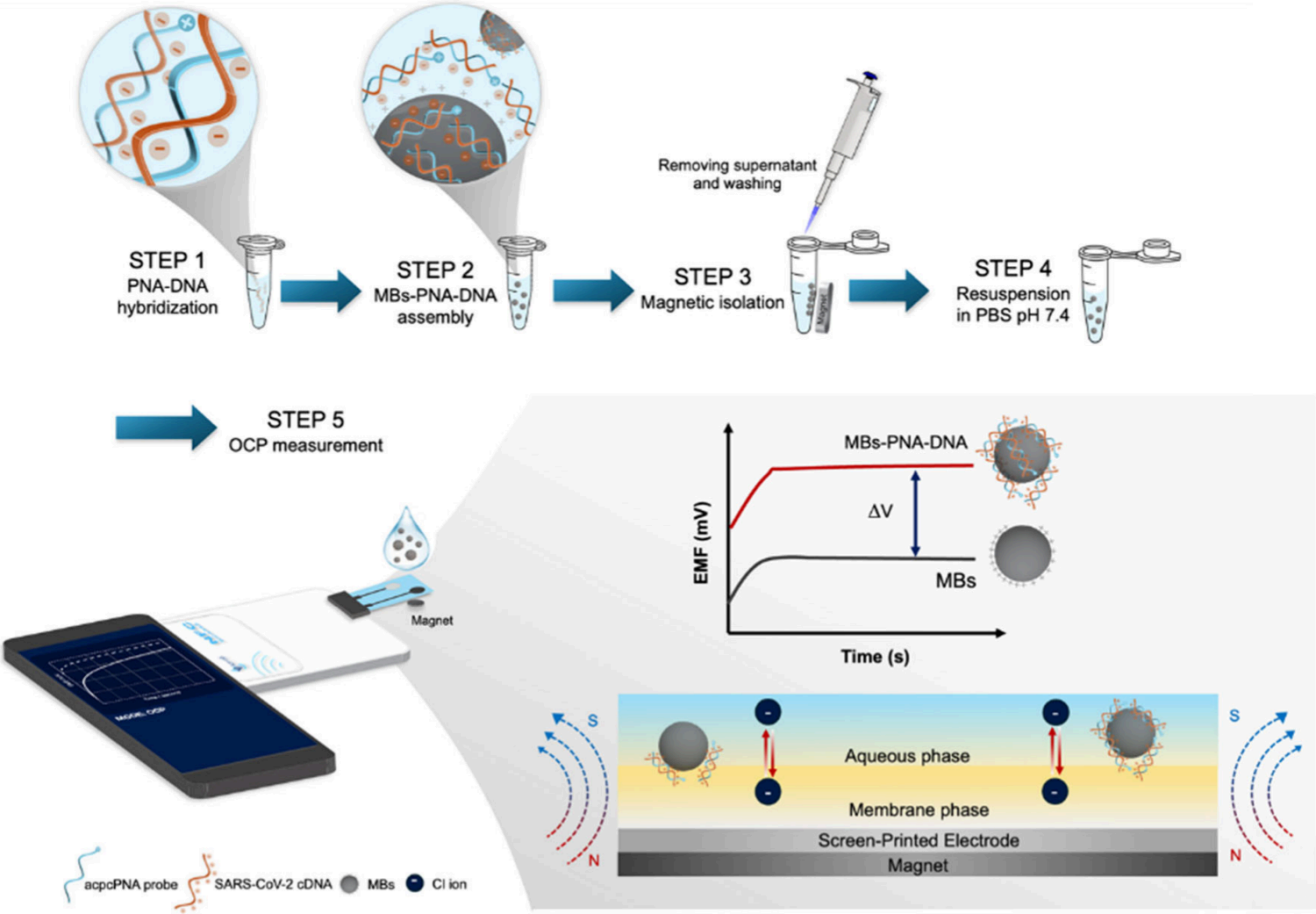
Potentiometric detection system for COVID-19 diagnosis using acpcPNA and magnetic beads.

According to the quasi-steady-state response model, the concentration of acpcPNA in the membrane phase can be given by equation^26^

1 $$\left[NA^{z-}\right]=\frac{D_{aq,NA}\delta_{m}}{D_{m,NA}\delta_{aq}}c_{NA,bulk}$$

where *D*_*aq*_ *,*_*NA*_ represents the diffusion coefficients of the acpcPNA in the aqueous phase and *D*_*m,NA*_ represents the diffusion coefficients of the acpcPNA in the membrane phases. Similarly, the thicknesses of the diffusion layer in the aqueous and membrane phases are shown as *δ*_*m*_ and *δ*_*aq*_, respectively. *δD* is expressed by eq 2 ^27^

2 $$\delta_{D}\approx 1.84{\left(\mu_{0}\rho Rv^{2/3}D^{4/3}\right)}^{1/3}{\left(\chi_{m}CB^{2}\right)}^{-1/3}$$

where *μ*_0_ is the magnetic permeability of free space, ρ is the constant density of a Newtonian fluid, *v* is the solvent viscosity, *D* is the diffusion constant of the magnetic species, *χ*_*m*_ is the molar magnetic susceptibility of the species involved, *C* is the bulk concentration of magnetic species, and *B* is the magnetic field strength.

### Optimization of Membrane Components

The polymeric membranes for ISEs typically consist of PVC, a plasticizer, and an ion exchanger.^28^ At first, membrane compositions varying in PVC, bis­(2-ethylhexyl) sebacate (DOS), and 2-nitrophenyl octyl ether (NPOE) as plasticizers and potassium tetrakis­(4-chlorophenyl)­borate (KTClPB) as the ion exchanger were used for the ISE membrane optimization in the presence of 1.0 mg/mL aminated magnetic beads, 10 μM PNA, and 10 μM target DNA to maximize the potentiometric signal. As a control experiment, a bare SPE electrode without the polymeric membrane modification was also tested. The polymeric membrane components and compositions are presented in Table S2. As a starting point, the SPEs were coated with three layers of the membrane (3 × 2 μL) based on our previous study of the construction of the ISEs fabricated using the same graphite type and graphite/carbon ink ratio.^29,30^ We hypothesized that KTClPB as an ion exchanger including an anionic site may facilitate the adsorption of the aminated MB-acpcPNA assembly in the membrane phase. Therefore, KTClPB was added to the membrane cocktail to investigate the effect of the anion exchanger in the membrane phase on the extraction of the analyte. For a comparative study, polymeric membrane cocktails without KTClPB were also prepared and used to evaluate the sensitivity of the electrodes as a control experiment.

As can be seen in Figure 2A,B, the electrode modified with membranes containing KTClPB showed lower potentiometric responses compared with the electrodes modified with the membrane prepared in its absence. For instance, the membrane cocktail including 1% KTClPB (M3 and M9, Table S2) showed slopes of −37.5 ± 1.0 and 13.0 ± 6.3 mV for the membranes prepared with DOS and NPOE plasticizers, respectively. The corresponding slopes for the membranes prepared without KTClPB were −38.2 ± 2.3 and 29.4 ± 2.8 mV for DOS and NPOE, respectively. On the contrary, Mou et al. showed that the anion exchange process at the sample–membrane interface between aptamers was obtained due to the presence of a polyion in the membrane phase.^31^ This could be due to the fact that the ion exchangers leached into the sample solution, resulting in a decreased signal.

**Figure 2:**
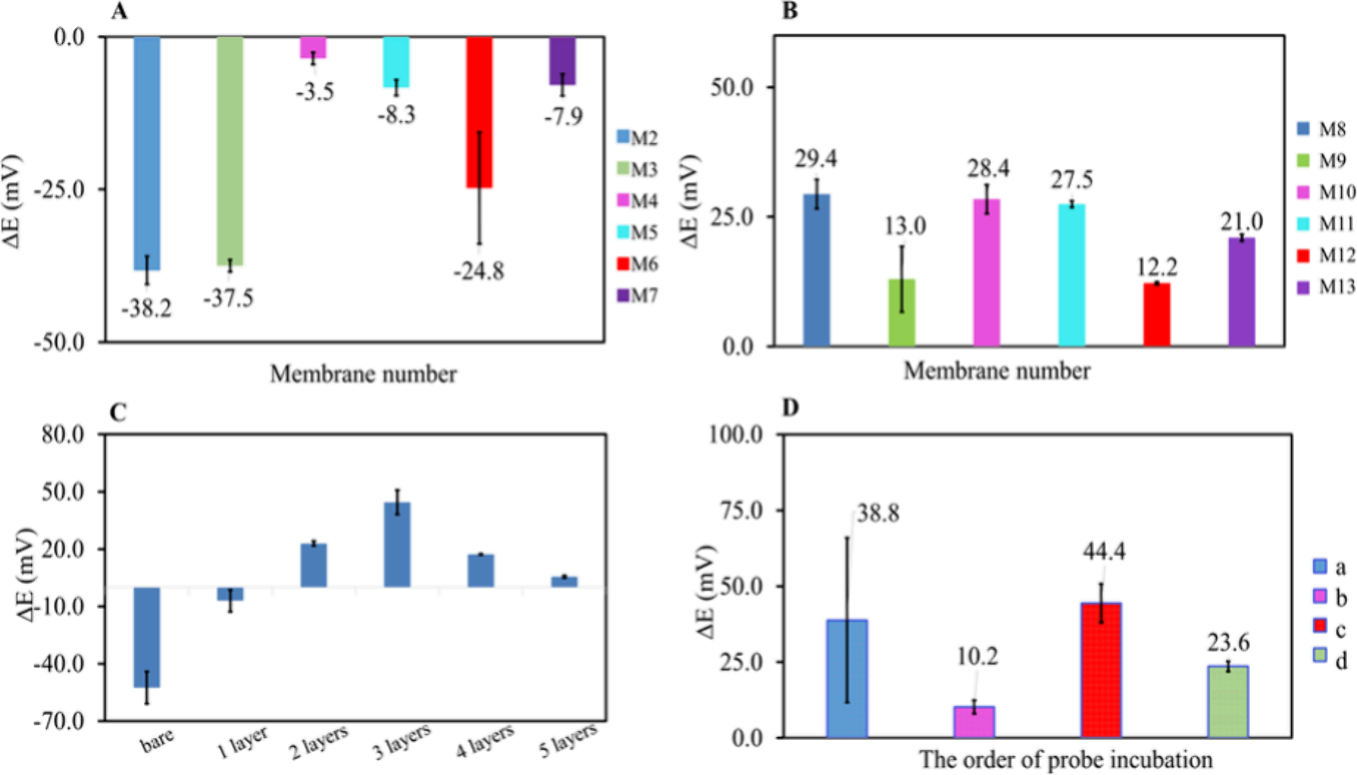
Potential difference for (A) different membrane compositions M2–M7, (B) different membrane compositions M8–M13 (three replicate electrodes; for membrane compositions, see Table S2), and (C) different numbers of polymeric membrane layers on the SPEs (five replicate electrodes). (D) (a) One-hour of NH_2_-MBs and acpcPNA incubation, followed by 1 h of DNA incubation, (b) 1 h of NH_2_-MBs and DNA incubation, followed by 1 h of acpcPNA incubation, (c) 1 h of acpcPNA and DNA incubation, followed by 1 h of NH_2_-MBs incubation, and (d) 1 h of NH_2_-MBs and acpcPNA and DNA incubation, simultaneously.

The effects of the plasticizer on the potentiometric response slope of the modified electrodes were also investigated. According to the results shown in Figure 2A,B, the electrodes modified with a polymeric membrane consisting of DOS as a plasticizer showed a negative potentiometric response value (−38.2 ± 2.3 mV) while the electrodes modified with a polymeric membrane consisting of NPOE showed a positive potentiometric response value (29.4 ± 2.8 mV). This could be due to the more polar nature of NPOE compared to that of DOS in which a polar phase of aqueous solution containing the probe-target assembly limits any charged species (the probe-target assembly) transfer to the nonpolar membrane phase consisting of DOS.^32^ These results indicate that potentiometric selectivity can be achieved if the target analyte transfers from the aqueous solution phase to the membrane phase more than other ions present in the sample.^33,34^ As a control, the membranes without plasticizers were also prepared using only PVC in THF. The electrode response was obtained at 24.3 ± 3.6 mV whereas the bare SPE without a membrane had a potential signal difference of −30.4 ± 31.2 mV. The plasticized PVC leads to the orientation of the polar C–Cl bonds in an electric field, decreasing its intensity and increasing the dielectric permittivity, whereas the rotation of the C–Cl bond around the C–C bond of the polymer backbone is not able to orient (is frozen) in pure PVC (without plasticizer).^35^

The polymeric membrane consisting of 33.3% PVC and 66.7% NPOE (M8, Table S2) gave the highest response, which is in agreement with the literature.^36^ Therefore, this membrane composition was chosen to fabricate the biosensor due to its highest signal (29.4 ± 2.8 mV) among those of others materials.

### Effect of the Number of Membrane Layers

The polymeric membrane thickness as the diffusion layer on the SPE surface directly influences the flux between the aqueous phase and the membrane phase, resulting in a change in potential responses.^26,37^ In addition, drop-casting a sufficient amount of membrane material is crucial to homogeneously covering the electrode surface; therefore, the number of membrane layers was optimized.^38^ It was found that low volumes of the membrane cocktail (1 × 2 μL and 2 × 2 μL) coated on SPEs were not suitable based on their low sensitivities (−7.0 ± 5.7 and 22.9 ± 1.3 mV, respectively) compared to that of the electrodes modified with 6 μL (3 × 2 μL) of membrane solution (44.4 ± 6.4 mV) (Figure 2C). However, the electrodes modified with 8 μL (4 × 2 μL) and 10 μL (5 × 2 μL) exhibited lower responses (17.2 ± 0.3 and 5.5 ± 0.6 mV, respectively), which could be due to the thickness of the membrane layer. As a control, bare electrodes without the polymeric membrane were also tested, and the potentiometric response slope was found to be −52.5 ± 8.4 mV. Therefore, the number of membrane layers needed to modify the SPE surface for further experiments was chosen as three (3 × 2 μL). These findings are also compatible with our previous procedures to fabricate the ISEs.^20,21,29^

According to our previous study, we modified the SPE-ISE surface with carbon black nanomaterial as an intermediate layer for enhancing the potentiometric response due to the prevention of an aqueous layer between the membrane and the transducer.^39,40^ The response behavior of the sensor was also investigated in the presence of carbon black using the membrane, including PVC and NPOE (M8, Table S2). Therefore, we followed a similar procedure to modify the electrode surfaces. Two layers (2 × 2 μL) of 5 mg mL^–1^ carbon black suspension were drop-cast on the SPEs, followed by incubation of three layers of the membrane (M8, Table S2). It was observed that the electrodes modified with carbon black demonstrated a potential response of 9.1 ± 2.5 mV, indicating a lower response compared to that of the electrodes without carbon black (44.4 ± 6.4 mV). We hypothesized that this could be due to the insulator behavior of carbon black that blocks the path for electron transport, hindering the transport of ion fluxes.^41^ Therefore, no carbon black layer was used in the parameter optimization of the sensor.

### Optimization of the acpcPNA Probe Incubation Order

We hypothesized that variations in the potential response of the electrodes might occur once the acpcPNA probe is introduced to either positively charged NH_2_-MBs or negatively charged DNA due to a conformational change in the target-binding acpcPNA. To test this hypothesis, four different orders of mixing among MBs, acpcPNA, and DNA were tested: (1) 1 h incubation of NH_2_-MBs and DNA, followed by 1 h incubation with acpcPNA, (2) 1 h incubation of NH_2_-MBs and acpcPNA, followed by 1 h incubation with DNA, (3) 1 h incubation of acpcPNA and DNA, followed by 1 h incubation with NH_2_-MBs, and (4) 1 h incubation of NH_2_-MBs and acpcPNA and DNA. In all experiments, the concentrations of acpcPNA, DNA and NH_2_-MBs were kept constant at 10 μM, 10 μM, and 1 mg mL^–1^, respectively.

According to the results (Figure 2D), it was found that 1 h acpcPNA and DNA incubation to first form the acpcPNA-DNA assembly followed by 1 h incubation with NH_2_-MB (the fourth condition) gave the best potential response compared to others. The first incubation condition, 1 h incubation of NH_2_-MBs and acpcPNA followed by 1 h incubation with DNA, gave the highest standard deviation since the interaction between acpcPNA and MBs is not favorable, resulting in the detachment of PNA from the MBs surface. This might be due to the electrostatic repulsion between the positively charged lysine residues at the N-terminus of the acpcPNA probe and the positively charged MB surface. Under the second condition, 1 h incubation of NH_2_-MBs and DNA leads to strong adsorption of DNA on the MBs surface. Under the third condition, NH_2_-MBs, the PNA probe, and DNA were incubated together at the same time, resulting in the hybridization of PNA-DNA while some negatively charged DNA was attached to the positively charged MBs, decreasing its ability to form PNA-DNA hybrids. The fourth condition gave the best potential response because the PNA probe was allowed to hybridize with the DNA target in an effective orientation in the first step (Figure 2D-c). Then, this PNA-DNA duplex was properly bound to positively charged MBs. Therefore, 1 h incubation of acpcPNA and DNA followed by 1 h incubation with NH_2_-MBs was chosen as the optimum incubation order for further experiments. These results signify that the operational sequence significantly affects the sensing performance.

The target-binding events could also affect the charge or charge density change of acpcPNA-DNA assembled on the MBs. Therefore, the zeta potentials of the aminated MBs (in 10 mM PBS at pH 7.4) and aminated MBs-acpcPNA-DNA assembly were evaluated at a concentration of 1 mg mL^–1^. The zeta potential of NH_2_-MBs was 2.9 mV and changed to −10.5 mV after incubation with acpcPNA-DNA due to the presence of negative charges in DNA. PNA has an advantage over DNA probes in that it prevents nonspecific adsorption via electrostatic attraction owing to its neutral charge. It is clear that the incubation order of the probe during the sensor surface modification has a significant effect on the signal response and ion-exchange process because of the reconfiguration of probe-target-magnetic nanoparticle binding. These results are also in agreement with the previous experiments on the incubation sequence of MBs-probe-target.

### Optimization of Magnetopotentiometry for DNA Detection

Here, the neutrally charged acpcPNA serves as the recognition element while the aminated-MBs-acpcPNA-DNA was the transduction indicator for quantitative detection in a direct manner. In addition, only aminated-MBs produce a potentiometric signal in the membrane phase, which could be due to the redistribution of ionic species on the sample–membrane interface. A signaling output was generated owing to folding of the acpcPNA around the DNA target and the interaction between NH_2_-MBs. Therefore, the magnetic field strength and quantities of magnetic beads were optimized in addition to polymeric membrane components and the number of membrane layers to obtain an enhanced potentiometric response. Various quantities of magnetic beads including 0.25 mg mL^–1^, 0.5 mg mL^–1^, 1.0 mg mL^–1^, 1.5 mg mL^–1^, and 2.0 mg mL^–1^ were incubated with the acpcPNA-DNA complex for 1 h to test the effect of the quantities of magnetic beads on the potential response. As can be seen in Figure 3A, the biosensor’s response increased and reached the highest potentiometric response (62.4 ± 10.5 mV) with 1.5 mg mL^–1^ MBs. Therefore, 1.5 mg mL^–1^ MBs was chosen as the optimum quantity of MBs.

**Figure 3:**
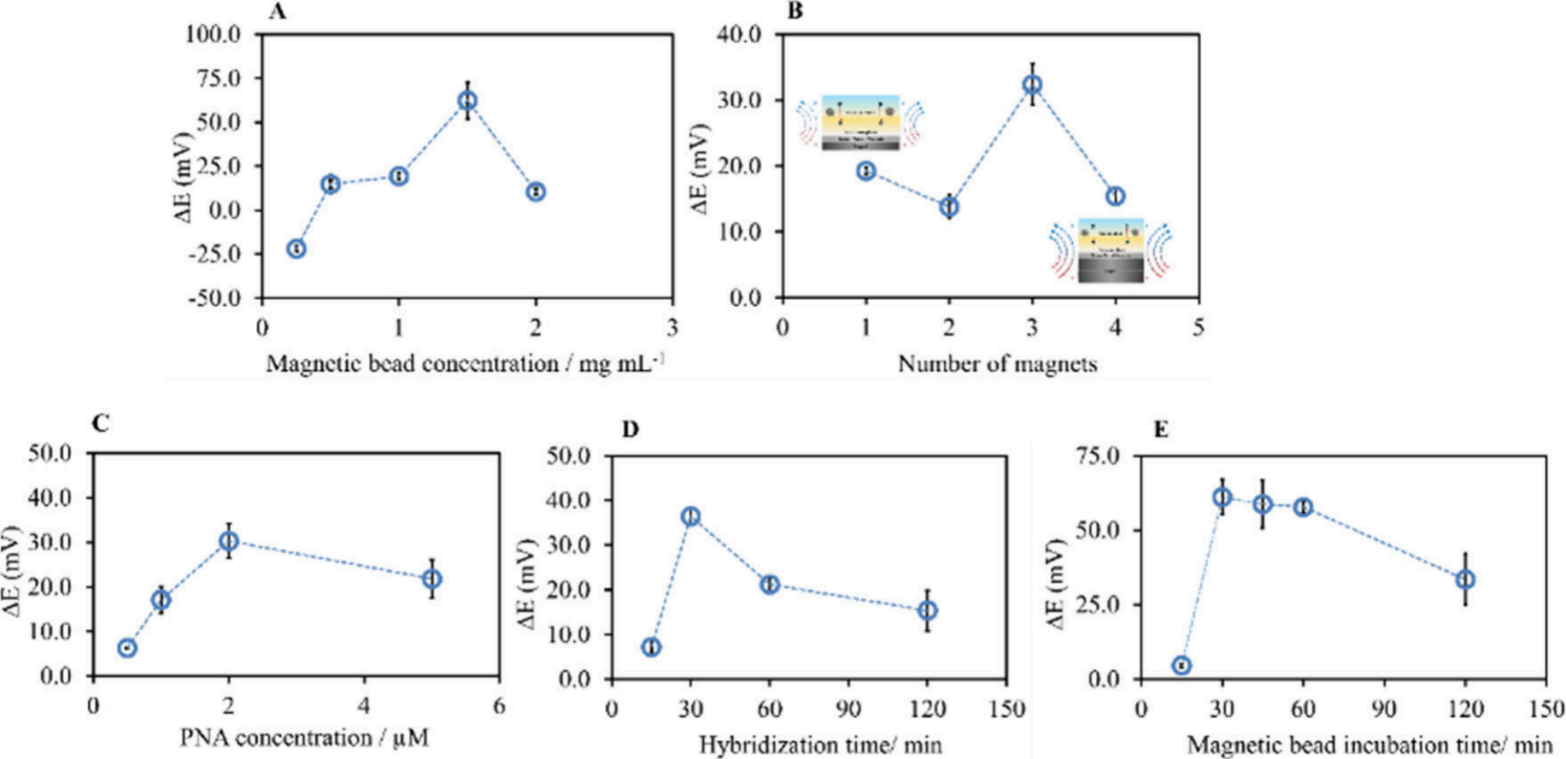
(A) Potential changes in MB-PNA-DNA with different magnetic field strengths by using different numbers of magnets. (B) Potential responses of various amounts of MBs on the SPEs. Error bars represent one standard deviation for four measurements. Optimization of (C) the PNA probe concentration and (D) different hybridization times (15, 30, 60, and 120 min) of the PNA biosensor with the target DNA. (E) Different incubation times (15, 30, 45, 60, and 120 min) of magnetic beads with PNA-DNA assembly. Error bars represent one standard deviation for three measurements.

Another parameter that affects the magnetic field is the magnetic force, which is controlled by the number of magnets. The MB-acpcPNA-DNA complex was adsorbed onto the sensing surface due to the presence of magnetically driven diffusion fluxes controlled by the strength of the magnetic field. Thus, the number of magnets adopted to generate the magnetic field during potentiometric measurements was tested using one to four magnets. Since there is a diffusion layer thickness generated by the polymeric membrane, the potential responses were enhanced by the increased number of magnets placed on the back side of the working electrode during potentiometric measurements owing to the enhanced magnetic field. The maximum potentiometric response toward the MB-acpcPNA-DNA network was obtained in the presence of three magnets (Figure 3B).

### Optimization of the Probe Concentration and Incubation Times

The acpcPNA probe concentration, acpcPNA-DNA hybridization time, and subsequent incubation time of the acpcPNA-DNA complex with MBs (1.5 mg mL^–1^ in 10 mM PBS) were also optimized. First, different acpcPNA concentrations including 0.5, 1.0, 2.0, and 5.0 μM were incubated with the target DNA (1 nM in 10 mM PBS at pH 7.4) to find the optimal performance of the biosensor. According to Figure 3C, the biosensor showed the highest signal response of 30.3 ± 3.8 mV at 2.0 μM acpcPNA probe concentration. Then, the acpcPNA-target DNA hybridization time was evaluated by incubation of 2.0 μM PNA with SARS-CoV-2 DNA for 15 min, 30 min, 1 h, and 2 h. The potentiometric response reached the highest value when the hybridization time was 30 min (Figure 3D).

Next, the incubation time of the magnetic beads (1.5 mg mL^–1^) with the acpcPNA-DNA complex was varied (15 min, 30 min, 45 min, 1 h, and 2 h). The increase in incubation time resulted in increasing response and reached a plateau during the period of 30 min to 1 h, and then the response decreased after that. Hence, the optimal acpcPNA concentration and hybridization time were 2.0 μM and 30 min, respectively, while the optimal quantity of magnetic beads and incubation time were 1.5 mg mL^–1^ and 30 min, respectively.

### Analytical Performance of Potentiometric Devices

The acpcPNA-based biosensor was applied to detect SARS-CoV-2 DNA using an N-gene synthetic DNA oligonucleotide (see sequence in Tabls S1) as a model at various concentrations in 10 mM PBS (pH 7.4). The linear potentiometric responses of the biosensor to the logarithm of DNA concentration are presented in Figure 4. Under optimized conditions, SARS-CoV-2 DNA was detected over a broad linear range of 1.0 × 10^–15^–1.0 × 10^–11^ M (Figure 4A). Five blank measurements were made, and the standard deviation of the blank response was determined to be 1.7 mV. The LOD was calculated to be 4.9 × 10^–16^ M using the equation LOD = 3.3σ/*S*, where σ is the standard deviation of the blank signal and *S* is the slope of the calibration curve. After the washing process, the biosensor responses were also recorded to investigate the reusability (Figure 4B). However, it was observed that there was a smaller potential response difference after the washing steps (64.0 ± 1.3 mV) compared to the first measurement (80.2 ± 5.8 mV), and the sequential addition of a 100 fM SARS-CoV-2 DNA sample to the electrode surface gave a further signal decrease, demonstrating that the developed sensors are suitable for single use. In addition, the larger standard deviations observed at the lowest DNA concentrations can be attributed to stochastic molecular transport and Poisson distribution effects when the number of target molecules approaches the detection limit, which leads to a variability in hybridization events at the sensor surface.

**Figure 4:**
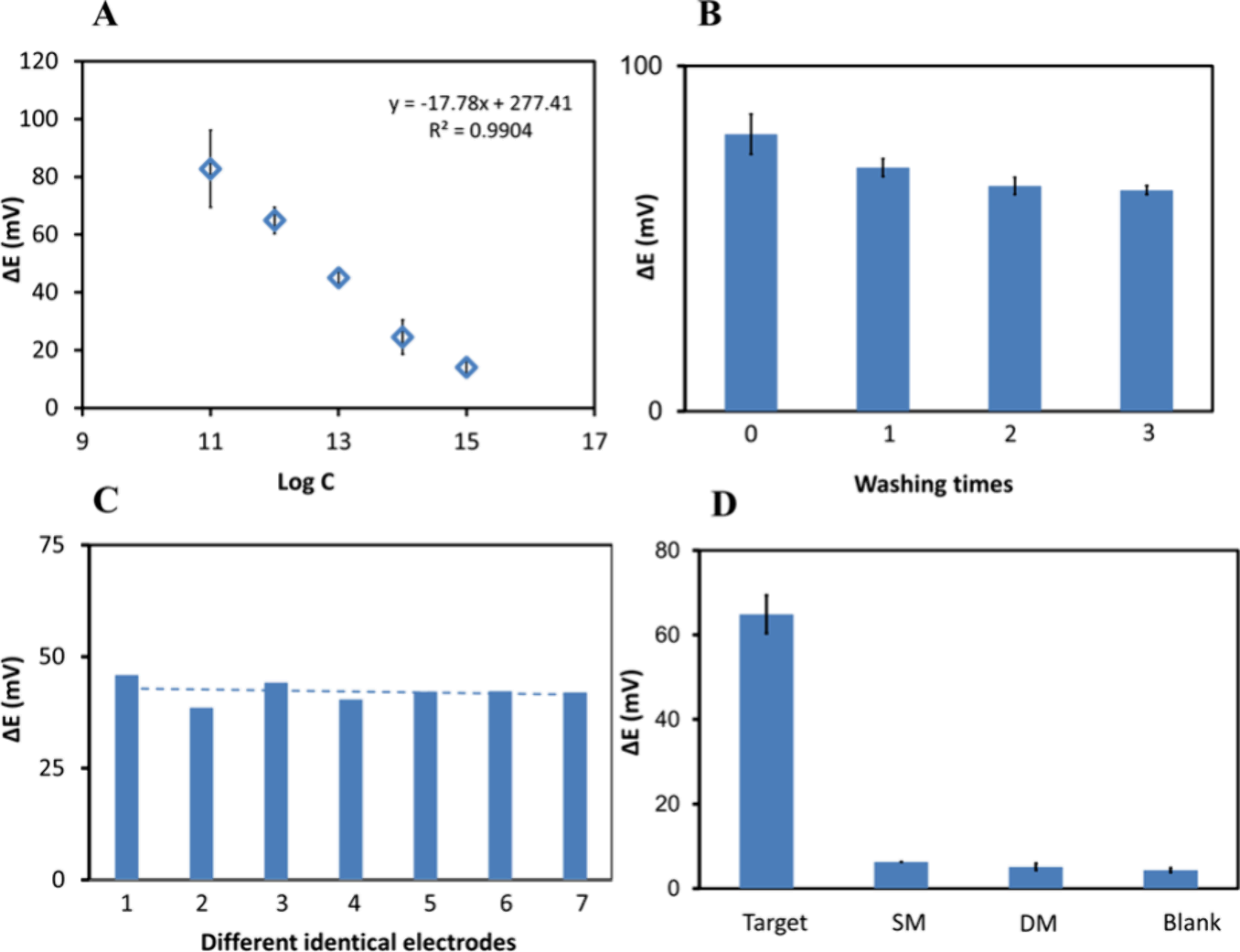
(A) Potentiometric responses of MBs-PNA-DNA in the presence of 1.0 × 10^–11^–1.0 × 10^–15^ SARS CoV-2. (Error bars represent the standard deviation of three independent measurements.) (B) Potential responses of the polymeric membrane-modified SPEs (A) before and (B) after washing steps using 10 mM PBS at pH 7.4. (Error bars represent the standard deviation for five replicate and three replicate measurements for the calibration curve and washing times, respectively.) (C) Biosensor’s responses to (1 pM) target and single-, double-base mismatched DNAs. (Error bars: SD, *n* = 3.) (D) Δ*E* (mV) responses of seven independently prepared biosensors for the detection of 100 fM target DNA.

Since the reproducibility of different sensors when prepared on a mass-production scale is important, the developed biosensors were tested using seven independently prepared biosensors. The potentiometric responses to 100 fM target DNA were recorded, and the relative standard deviation was found to be 5.6%, indicating good reproducibility of the biosensor (Figure 4C). The stability of the biosensors was also evaluated to ensure long-term storage of the biosensor. The electrodes were tested weekly over 2 months. There was no potential change observed due to the preparation and application of biological reagents just before the analysis. Therefore, the plasticized PVC membrane electrodes exhibited long-term stability (up to 2 months), in good agreement with previous reports.^42^ Cross-reactivity was also evaluated to investigate the specificity of the developed biosensor. The specificity of the biosensor was tested using 1.0 pM target SARS-CoV-2, 1.0 nM single-base (SM) SARS-CoV-2 DNA, and double-base mismatched (DM) SARS-CoV-2 DNA (sequences in Table S1). As shown in Figure 4D, the developed biosensor exhibited good selectivity and did not show significant potential changes with the other DNA sequences. It was observed that the biosensor demonstrated a high specific response to a low concentration of target SARS-CoV-2 DNA while the potentiometric responses were much lower for even higher concentrations of mismatched bases. Thus, the developed magnetically driven potentiometric biosensor can sensitively and selectively detect the target molecules due to the high specificity of the acpcPNA probe.

### Proof-of-Concept Detection of SARS-CoV-2 on a Smartphone

The developed biosensor along with a near-field communication (NFC) potentiometer operated by a smartphone was used as a proof-of-concept experiment. NFC includes a wireless communication setup and is employed with the use of a smartphone, making it applicable for POC applications. The plasticized PVC-modified SPE was connected to the NFC potentiometer printed circuit board. The open circuit potential was determined in the presence of a blank and the sample in PBS using the NFC potentiometer. The potentiometric response was shown as a video. The amounts of SARS-CoV-2 RNA have been reported as 1.0 × 10^4^ to 1.0 × 10^8^ copies/mL in patient saliva, which is equal to 1.0 pM to 10.0 nM. Therefore, SARS-CoV-2 DNA was serially diluted with human saliva to 1.0–10.0 pM. The saliva samples containing various concentrations of SARS-CoV-2 DNA were used to construct a calibration curve. Under optimal conditions, the developed biosensor was applied to detect SARS-CoV-2 using a standard addition method. The results are in good agreement with the added values, showing the recovery values in the range of 96.0–98.4% and confirming the practicability of the biosensor (Table 1). Using the IUPAC criterion (LOD = 3.3σ/*S*), the LOD in human saliva was calculated to be 4.3 × 10^–13^ M, slightly higher than the LOD obtained in buffer (4.9 × 10^–16^ M) due to the matrix effects. The standard deviation of the blank response was determined to be 2.0 mV by using three replicate measurements. The results showed that the portable sensing platform consisting of a smartphone-based NFC potentiometer can be used for the detection of SARS-CoV-2 at the POC. The comparison of the developed biosensor with other sensors in the literature is presented in Table 1.

**Table 1:** Determination of the Target in Human Saliva

Number	Added (pM)	Found (pM)^a^	Recovery (%)
1	1.00	0.970 ± 0.50	97.0
2	5.00	4.92 ± 0.60	98.4
3	10.0	9.60 ± 0.50	96.0

a Average of three measurements.

## Conclusions

The detection techniques with high sensitivity generally require laborious procedures or greater concentrations of viral genetic material for detection. This study presents a novel PNA-based device for potentiometric biosensing, which has the advantages of high specificity and stability features, using two steps for the rapid diagnosis of COVID-19 in a direct manner. The device provided various unique properties. The electrochemical sensing platform consists of a plasticized membrane coated solid-contact electrode assisted by amine-functionalized magnetic nanoparticles and synthesized acpcPNA probes to detect SARS-CoV-2 DNA. Due to the incorporation of electrically neutral PNA, the developed biosensor showed high sensitivity toward SARS-CoV-2 with an LOD of 0.49 fM and was successfully applied to detect SARS-CoV-2 DNA in human saliva. The developed sensor features acpcPNA as an electrostatically neutral probe that does not interact nonspecifically with charged molecules, thus offering new modes of DNA detection. The developed biosensor offers several advantages compared to many previous reports due to its simplicity of operation, applicability in complex biofluids, and high stability owing to the use of PNA probes and occurrence of the hybridization process outside of electrode zone real time. The sensitivity of the sensor at the low femtomolar level suggests that it does not require an amplification process; however, a reverse transcription procedure is needed to obtain SARS-CoV-2 cDNA. There is preliminary evidence showing high stability overstorage (2 months) of the sensor and PNA itself. Further development is currently being pursued to streamline the operation by combining the hybridization and incubation steps with the use of microfluidics technology. The sensing platform could be adapted to detect other viruses in future pandemics.

## Supplementary Material



## Notes

Materials, electrode fabrication, membrane preparation, synthesis of the acpcPNA probe, electrochemical measurements, characterization of surface morphology, sample preparation, verification of acpcPNA synthesis, characterization of the modified biosensor, and cost analysis of the proposed biosensor (PDF)
