# Supplementary material for: Smartphone-Based Peptide Nucleic Acid (PNA) Probe-Assisted Potentiometric Biosensor for Point-of-Care Testing of SARS-CoV‑2 Nucleic Acid
Source: Anal Chem. 2026 May 5;98(19):14382–90. doi: 10.1021/acs.analchem.6c00766 (PMC13191720; doi:10.1021/acs.analchem.6c00766)

A Smartphone-Based Peptide Nucleic Acid (PNA) Probe-Assisted Potentiometric Biosensor for Point-of-Care Testing of SARS-CoV-2 Nucleic Acid

Tugba Ozer [a,b]*, Sakda Jampasa [c], Atchara Lomae [d,e], Nipapan Ruecha [f], Osman Sagdic [g], Tirayut Vilaivan [h], Charles S. Henry [b,f], Orawon Chailapakul [j]*

[a] *Department of Bioengineering, Faculty of Chemical-Metallurgical Engineering, Yildiz Technical University, Istanbul, 34220, Türkiye*

[b] *Department of Chemistry, Colorado State University, Fort Collins, Colorado, 80523, USA*

[c] *Futuristic Science Research Center, School of Science, Walailak University, Nakhon Si Thammarat, 80160, Thailand*

[d] *Center of Excellence for Trace Analysis and Biosensor, Prince of Songkla University, Songkhla, 90110, Thailand*

[e] *Division of Physical Science, Faculty of Science, Prince of Songkla University, Songkhla, 90110, Thailand*

[f] *Metallurgy and Materials Science Research Institute, Chulalongkorn University, Bangkok, 10330, Thailand*

[g] *Department of Food Engineering, Faculty of Chemical-Metallurgical Engineering, Yildiz Technical University, Istanbul, 34220, Türkiye*

[h] *Organic Synthesis Research Unit, Department of Chemistry, Faculty of Science, Chulalongkorn University, Bangkok, 10330, Thailand*

j *Electrochemistry and Optical Spectroscopy Center of Excellence (EOSCE), Department of Chemistry, Faculty of Science, Chulalongkorn University, Bangkok, 10330, Thailand*

* Email: tozer@yildiz.edu.tr

* Email: orawon.c@chula.ac.th

Experimental section

Materials

High molecular weight polyvinyl chloride (PVC), potassium tetrakis(4-chlorophenyl)borate (KTClPB), 2-nitrophenyl octyl ether (NPOE), bis(2-ethylhexyl) sebacate (DOS), tetradodecylammonium tetrakis(4-chlorophenyl)borate, poly(butyl methacrylate-co-methyl methacrylate), KCl, and tetrahydrofuran (THF) were purchased from Sigma-Aldrich. Phosphate buffer saline (PBS) tablet, potassium ferricyanide and potassium ferrocyanide were purchased from Sigma-Aldrich (St. Louis, USA). The neodymium magnets (1/8″ × 1/8″ × 1/8″) were purchased from K&J Magnetics (Pennsylvania, USA). M-270 Amine Dynabeads with an average particle size of 2.8 μ were purchased from Thermo Fisher Scientific (Vilnius, Lithuania). All aqueous solutions were prepared using 18 Ω milli-Q water (Millipore, USA). All synthetic oligonucleotides were purchased from Integrated DNA Technologies (Iowa, USA). PNA probes Table S1 shows the DNA sequences in which all stock solutions were prepared in 0.1 M PBS, pH 7.4 and kept at -20 °C prior to use.

Table S1. The DNA sequences used in this work (the mismatched bases are illustrated by underline).

Name	Sequences (5' to 3')
Complementary target DNA (N gene)	GAT CGC GCC CCA CTG CGT TCT CCA TTC TGG TTA CTG CCA GTT G
Single-base mismatch DNA	GAT CGT GCC CCA CTG CGT TCT CCA TTC TGG TTA CTG CCA GTT G
Two-base mismatch DNA	GAT CGT GCC CCA CTG CGT TCT CCA TTC TGG GTA CTG CCA GTT G

Electrode fabrication

The device designs were generated using Adobe Illustrator CC (Adobe Systems, USA). TC303 graphite powder (Asbury Carbons, New Jersey, USA) and E3178 commercial carbon ink (Ercon Inc., Massachusetts, USA) were mixed at a ratio of 0.6:1 g w/w to obtain electrode composites.[23, 24] A 30 W Epilog Engraver Zing CO_2 laser cutter and engraver (Colorado, USA) was used to cut the poly(ethylene) terephthalate (PET) transparency sheet (3 M Minnesota, USA) for preparation the two-electrode design template. Then, the electrode composites were applied on the PET sheets with the use of a squeegee and fabricated electrodes were cured in an oven at 65 °C for 30 min. A PET sheet was placed between two layers of double-sided adhesive (3 M) for formation of a solution reservoir. The electrical resistance of the SPCEs was tested using a Fluke 187 multimeter (0.01 Ω accuracy).

Membrane preparation

A mixture of the ion selective membrane (ISM) components including 66.7% (w/w) of NPOE and 33.3% (w/w) of high molecular weight PVC (0.20 g) in 1.5 mL of THF was prepared. Several membranes consisting of different ratios of components were also prepared and tested as presented in Table S2. Six µL of the membrane cocktail was drop-cast on the working electrode zones and allowed to dry overnight at room temperature. The reference electrode membrane that includes 200 mg poly(butyl methacrylate-co-methyl methacrylate), 68 mg KCl, and 5 mg tetradodecylammonium tetrakis(4-chlorophenyl)borate in 1 mL THF was prepared according to our recent study.[25-27] The reference electrode was fabricated by successive drop-cast of 2 µL of Ag/AgCl ink (60/40 Sigma) and reference membrane on reference electrode zone, respectively. The solid-state ISEs were placed in a desiccator overnight to allow complete solvent evaporation. Schematic representation of electrode fabrication is shown in Figure S1.

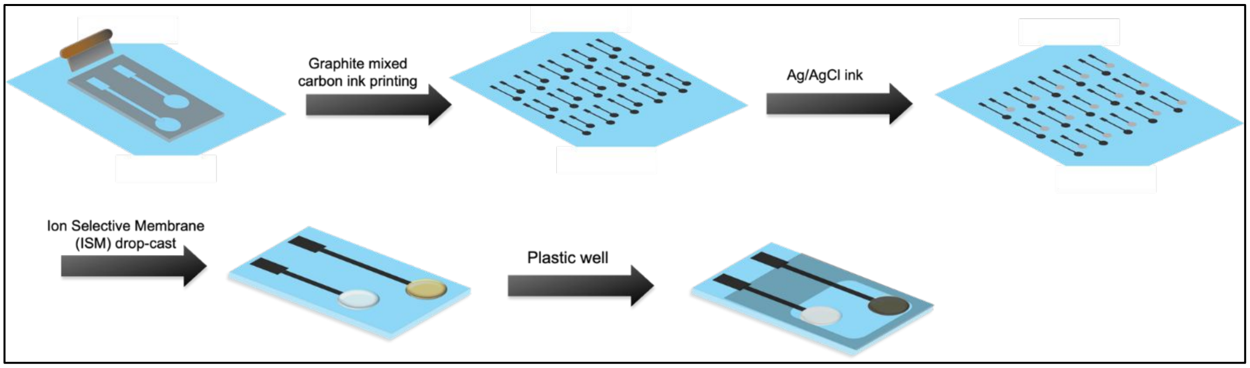

Figure S1. Schematic representation of electrode fabrication.

Table S2. Compositions of the ISE membranes.

Membrane	Plasticizer type	Plasticizer (wt.%)	PVC (wt.%)	KTClPB (wt.%)
M1	-	-	100	-
M2	DOS	66.7	33.3	-
M3	DOS	66.0	33.0	1
M4	DOS	65.3	32.7	2
M5	DOS	64.7	32.3	3
M6	DOS	63.3	31.7	5
M7	DOS	62.0	31.0	7
M8	NPOE	66.7	33.3	-
M9	NPOE	66.0	33.0	1
M10	NPOE	65.3	32.7	2
M11	NPOE	64.7	32.3	3
M12	NPOE	63.3	31.7	5
M13	NPOE	62.0	31.0	7

Synthesis of the acpcPNA probe

The sequences of the acpcPNA probe were designed to be complementary with SARS-CoV-2 (N gene type) and synthesized by the solid-phase peptide synthesis protocol according to the previous reports.[28, 29] The sequence of the acpcPNA probe (Ac-Lys-CAA CTG GCA GTA A-NH_2; written in the N to C direction) was designed to be complementary to the synthetic target SARS-CoV-2 DNA (N gene type).[16] The acpcPNA probe was synthesized via the solid-phase peptide synthesis protocol with the use of Fmoc-protected acpcPNA monomers (A^{Bz}, C^{Bz}, T and G) and (1*S*,2*S*)-2-amino-1-cyclopentanecarboxylic

acid (SS-ACPC) spacer at 1.5 μmol scale. After the desired sequence was assembled, the N-terminus was capped with Fmoc-Lys(Boc)-OPfp, followed by Fmoc group removal and N-terminal acetylation. The nucleobase sidechain protecting groups were removed using 1:1 concentrated aqueous ammonia-dioxane at 60 °C for overnight. Next, the acpcPNA was cleaved from the solid support using 95% trifluoroacetic acid (TFA) (500 μL, 30 min × 3) solution. The combined TFA was dried under a stream of nitrogen. The crude acpcPNA was precipitated by the addition of diethyl ether and purified by reverse-phase HPLC with a Water Delta 600TM system utilizing a C18 column and the gradient elution of 0.1% v/v TFA in an H_2O-MeOH. Finally, the acpcPNA was analyzed by MALDI-TOF MS on a Microflex MALDI-TOF mass spectrometer (Bruker Daltonics) employing α-cyano-4-hydroxycinnamic acid (CCA) as a matrix (*m/z* calcd. for $M \cdot H^+$ = 4583.9, found 4581.3), and by T_m experiments (T_m with complementary DNA = 72.5 °C, at 1 μM PNA and DNA in 10 mM sodium phosphate buffer pH 7.0 containing 100 mM NaCl).

Electrochemical measurements

Potentiometric open circuit potential (OCP) measurement was performed using PalmSens 4 potentiostat/impedance analyzer (PalmSens, USA) for optimization studies and analyzing the target analytes on the modified electrode surface without applying an external electric field. A two-electrode system was used for potentiometric measurements whereas three electrode system including the polymeric membrane modified working electrode was employed for electrochemical impedance spectroscopy (EIS). 10 μL of an analyte was drop-cast on the detection system at intervals after allowing the system to reach equilibrium until a stable signal was obtained, while the OCP was recorded in real-time. The detected OCP signal was normalized as $[\Delta V/V_0] = (V - V_0)/V_0$, where V is the detected real-time potential and V_0 is the initial potential.[28] All potentiometric measurements were carried out in 1.0×10^{-2} M PBS (pH 7.4) at room temperature. For EIS measurements, the impedance spectra were measured in 10 mM PBS within the frequency range (f = 1 MHz – 0.1 Hz) using an excitation signal amplitude of 100 mV and were fitted to equivalent circuits with Randles circuit (PalmSens, USA).

Characterization of Surface Morphology

The elemental compositions of the polymeric membranes in the presence and absence of MBs were recorded on a JEOL JSM-6500F field emission scanning electron microscope (FESEM) with EDAX an Oxford SDD EDS detector (Oxford Instruments, UK). X-ray photoelectron spectroscopy (XPS, PHI Physical Electronics PE-5800 X-ray Photoelectron Spectrometer, USA) was performed to examine the membrane elemental compositions and P 2p spectra in the presence of MBs-PNA. The extraction of the PNA into the polymeric membrane was confirmed by using a Fourier transform infrared spectrometer (FTIR, Thermo Nicolet iS-50 FTIR spectrometer, USA). The zeta potential of MBs in the presence and absence of PNA-DNA was measured using a Malvern Zetasizer Nano-ZS90 (ZEN3590, Malvern

Instruments Ltd., UK) in 10 mM PBS pH 7.4. The contact angle measurements were performed using a Drop Shape Analyzer Contact Angle Goniometer (Kruss DSA10, Germany).

Sample preparation

We chose saliva as a sample model for nasopharyngeal swab specimens to apply on our device since it could be collected easily from individuals and has increased clinical sensitivity owing to its high viral load. Saliva samples for medical research were obtained from volunteers in our laboratory who have been restrained from consuming food and water 30 minutes before the sample collection. Sample aliquots (200 μL) were spiked with the synthetic oligonucleotide corresponding to the N gene region of SARS-CoV-2 at the desired concentration.

Results and Discussion

Verification of acpcPNA synthesis

The identity of the synthesized acpcPNA probe was confirmed by MALDI-TOF mass spectrometry (Figure S2). According to the results, PNA was synthesized successfully with high purity.

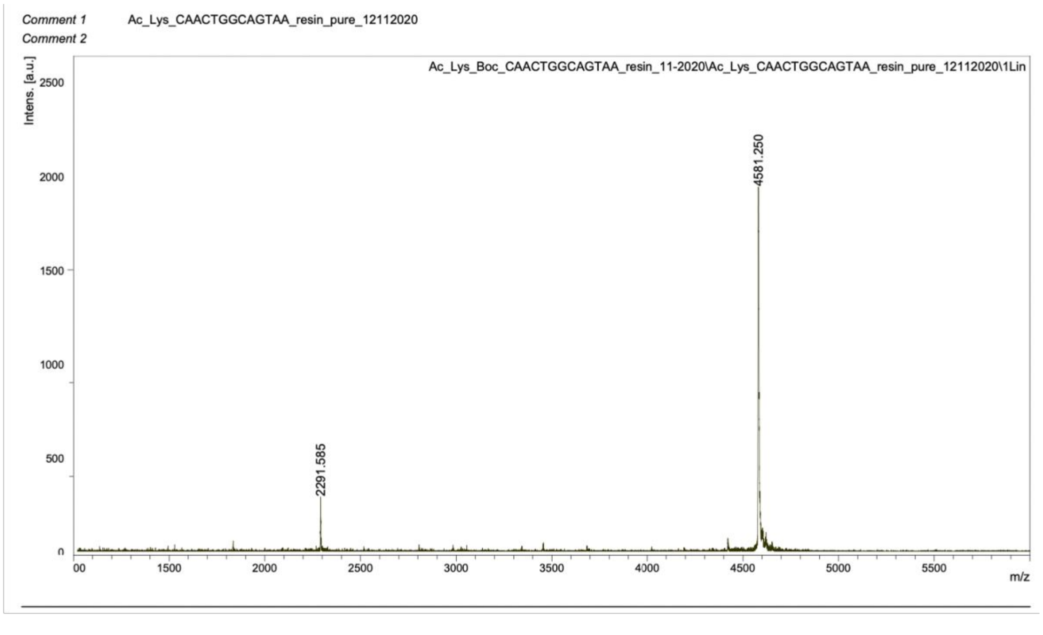

Figure S2. MALDI-TOF mass spectrum of the synthesized acpcPNA.

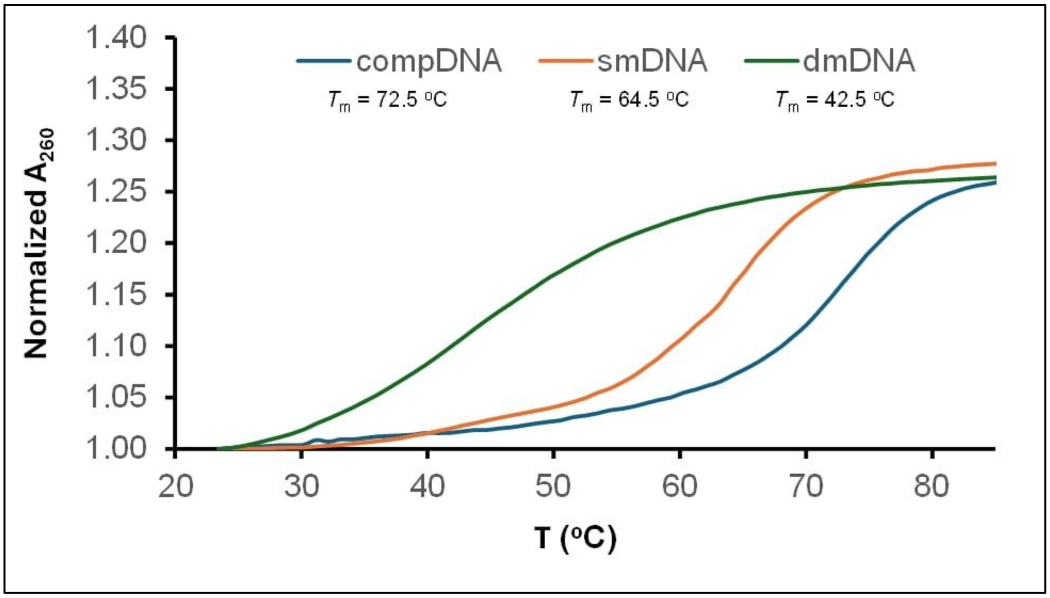

Figure S3. Comparison of T_m curves of hybrids of N-gene PNA (Ac-Lys-CAACTGGCAGTAA-NH_2) with complementary DNA (compDNA: dTTACTGCCAGTTG), single mismatched DNA (smDNA: dTTACTGTCAGTTG) and two mismatched DNA (dmDNA: dTTACTGTCAGGTG). Conditions: [PNA] = [DNA] = 1 μM in 10 mM sodium phosphate buffer pH 7.0, 100 mM NaCl.

The high sequence specificity of DNA recognition by PNA, especially the present pyrrolidinyl PNA system, has long been established. To confirm the mismatch discrimination ability of the N-gene PNA sequence used in this study, a melting temperature (T_m) experiment is performed by monitoring UV absorbance at 260 nm (A_{260}) as a function of temperature. The hybrid between the N-gene PNA and its complementary DNA sequence had a T_m of 72.5 °C. The presence of a single mismatched base at the middle position of the DNA strand resulted in a lower Tm of 64.5 °C. Introducing an additional mismatch base further decreases the *T*m to 42.5 °C. The higher T_m of the complementary PNA-DNA hybrid indicates greater stability than the mismatched hybrids, thereby explaining the large difference in the electrochemical response of the PNA probe towards complementary and mismatched DNA sequences.

Characterization of membrane thickness

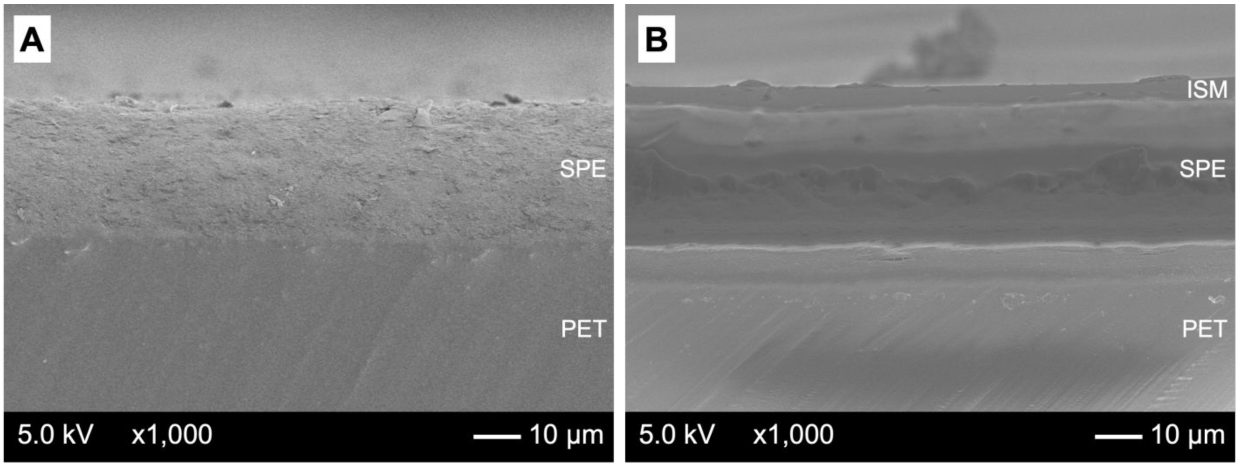

Figure S4. Cross-sectional SEM images of (A) bare electrode and (B) ISM coated electrode.

The thickness of the membrane layer was evaluated using cross-sectional field emission scanning electron microscope (FE-SEM) as shown in Figure S4. A well-defined multilayer structure consisting of the ISM, the SPE, and the PET substrate was clearly observed. The ISM coated on the electrode surface was observed as uniform, indicating successful membrane deposition without the presence of significant cracks or delamination. According to the SEM cross-sectional analysis, the thickness of the membrane layer was determined to be approximately 4.3 µm. This thickness is consistent with the sequential deposition of multiple membrane layers and confirms the controlled fabrication of the sensing interface.

Characterization of the modified biosensor

Field-emission scanning electron microscopy (FE-SEM) images and energy-dispersive spectrograms (EDS) were performed to demonstrate the accumulation of MBs-acpcPNA-DNA on the polymeric membrane modified electrode surface after extensive washing with 0.1 M PBS (pH 7.4). As can been seen in Figure S5, there is still MBs-acpcPNA-DNA on the electrode surface after washing steps, confirming the transfer of MBs-acpcPNA-DNA complex into the polymeric membrane.

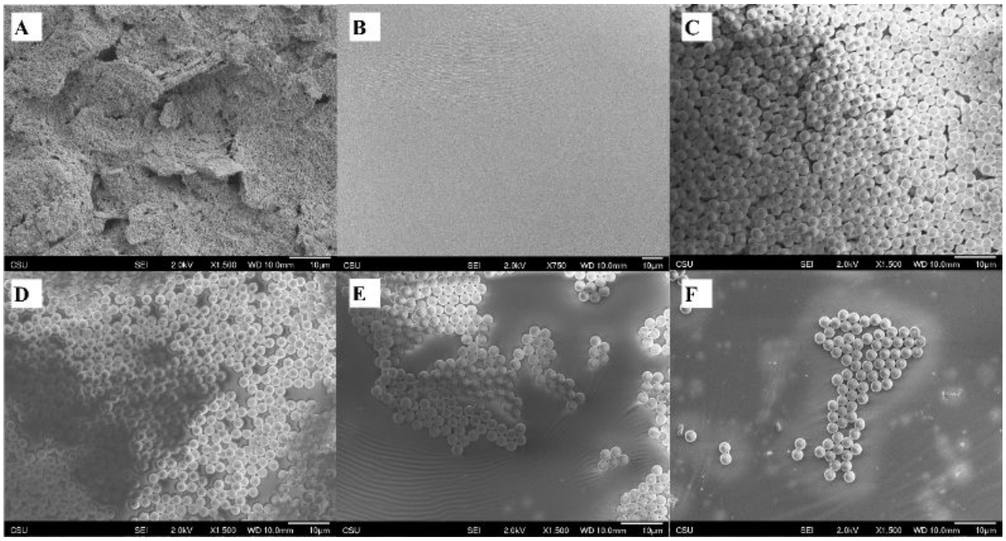

Figure S5. SEM images of (A) bare SPE, (B) PVC membrane coated SPE, in the presence of (C) aminated MBs, (D) MBs-PNA-DNA, (E) after washing step once, (F) after washing three times.

X-ray photoelectron spectroscopy (XPS) and Fourier transform infrared (FTIR) spectrum of the electrode coated with polymeric membrane in the presence of MBs and MBs- acpcPNA-DNA complex on the surface indicated that hydrophilic DNA phosphate backbone was extracted into the hydrophobic polymeric membrane with as shown by the P 2p peak at 155.0 eV (Figure S5A) following the addition of MBs-PNA-DNA. XPS spectra of PVC-MBs and PVC-MBs-PNA-DNA showed the presence of nitrogen due to the amino groups on the MBs. On the contrary, the PVC without MBs showed no nitrogen singals. In addition, O-H stretching peaks can be observed 3349 cm^{-1} in the FTIR spectrum (Figure S5B). The hydrophilicity of the various electrode surfaces were evaluated by measuring the water contact angles (Figure S5D). The measured contact angles of the bare SPE and NPOE plasticized PVC modified SPE were 130.0° and 88.2°, respectively. This confirms the decrease in hydrophilicity of the SPE surface following the application of the polymeric membrane.

A potentiostat (Palmsens 4) was used to carry out the electrochemical impedance spectroscopy measurements. The polymeric membrane coated (M8, Table S2) working electrode was tested within 10^3 kHz-0.1 kHz with an amplitude of 50 mV to obtain the impedance spectra. In Figure S6, the electrode-membrane and membrane-aqueous interfaces are presented as the bulk membrane resistance R_m in series and geometric capacitance C_g in parallel, respectively, using the electrochemical impedance model two Randles-type equivalent circuits.[29, 30] The impedance data were fitted using one single Randles-type equivalent circuit in which the double layer capacitance, C_{dl}, is in parallel along with the charge-transfer resistance, R_{ct}. According to Figure S6, it was observed that both R_m and R_{ct} were increased once MBs-acpcPNA-DNA was introduced to the electrode surface. The resistance of the membrane-solution interface

was much higher than the electrode-membrane interface, which is in agreement with the literature findings.[31]

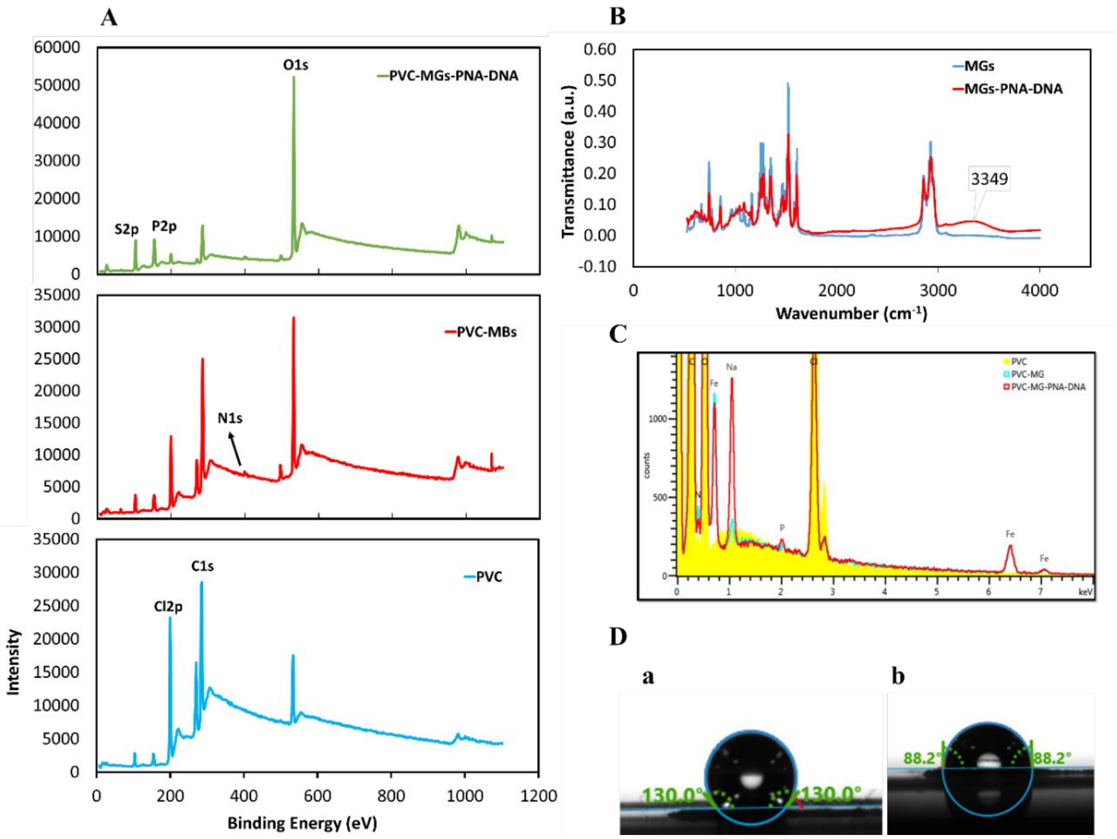

Figure S6. (A) XPS characterizations of PVC, Magnetic beads-PVC membrane, PNA/DNA/ Magnetic beads-PVC membrane on the electrode surfaces. (B) Fourier transform infrared (FTIR) spectra of the membrane without and with the addition of PNA-DNA. (C) Energy dispersive spectroscopy (EDS) without and with the addition of MGs and MGs-PNA-DNA. (D) The contact angles of (a) bare SPE (b) polymeric membrane consisting of PVC (33.3 wt.%) and NPOE (66.7 wt.%) coated SPE.

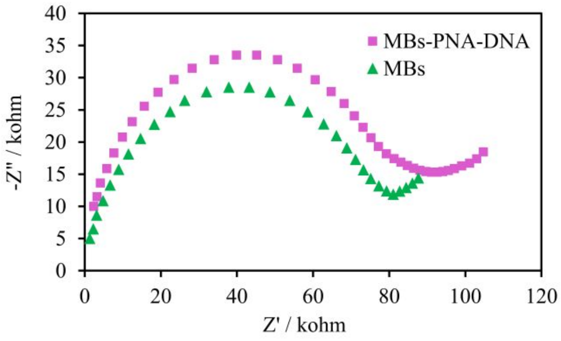

Figure S7. Impedance spectra (0.1–10^3 kHz, 50 mV amplitude) of the ISE sensor in 10.0 mM PBS (pH 7.4) before and after adding MBs-acpcPNA-DNA.

Table S3. Comparison of the developed biosensor with recently published reports that detecting SARS-CoV- 2 genetic material.

**Assay**	**Linear range**	**LOD**	**Stability**	**Analyte**	**Analysis time**	**References**
DPV	1.0 fM - 1.0 nM	0.38 fM	N/A	Human saliva	>1 hour	[12]
DPV	N/A	1.0 fM	N/A	PBS	N/A	[49]
DPV	1- 1×10^9 copies/µL	1 copies/µL	N/A	Nasopharyngeal swab	< 2 hours	[50]
DPV	0.1- 1000 pM	26 fM	N/A	Saliva and serum	4.5 hours	[51]
DPV	1.0×10^{-1}- 1.0×10^5 fM	8.1×10^{-2} fM	N/A	Artificial saliva	N/A	[52]
Potentiometric	1.0 fM-0.01 nM	0.49 fM	>two months	Human saliva	1 hour	This work

Cost analysis of the proposed biosensor

According to the World Health Organization (WHO), the ASSURED criteria defines typical values of US $1.00 to US $10.00 per test various rapid antigen tests to molecular assays as affordable. Moreover, the frequent use as weekly test (US $5.00) is also considered as low-cost compared to symptom-based tests to employ in low- to middle-income countries. Therefore, the cost of analysis is calculated and presented in Table S4.

Table S4. Cost analysis of the developed device.

	**Materials**	**Estimated cost/device ($)**
**Electrode**	Graphite powder	0.0014
	Carbon ink	0.015
	Ag|AgCl ink	0.009
	**subtotal**	**0.025**
**Membrane cocktail**	PVC	0.000068
	NPOE	0.00891
	**subtotal**	**0.008978**
**Reagents**	acpcPNA	0.110
	MBs	0.075
	PBS	0.0000161
	**subtotal**	**0.1850161**
**Total device cost**		**0.219**

Table of Contents

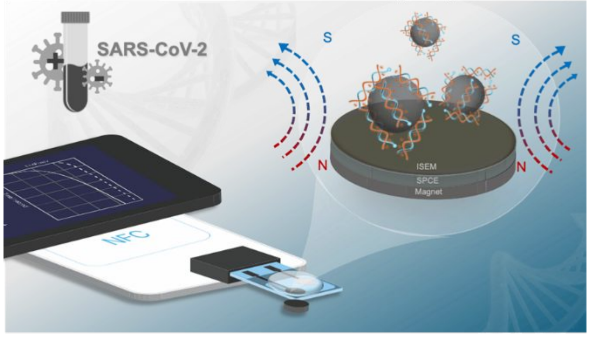

Supplement: Supplementary file 1 [file ac6c00766_si_001.pdf]
